# Supplementary material for: Engineering an in vitro retinothalamic nerve model
Source: Front Neurosci. 2024 May 21;18:1396966. doi: 10.3389/fnins.2024.1396966 (PMC11148348; doi:10.3389/fnins.2024.1396966)
Supplement: Supplementary file 1 [file Data_Sheet_1.pdf]

Supplementary Material

1 SUPPLEMENTARY FIGURES

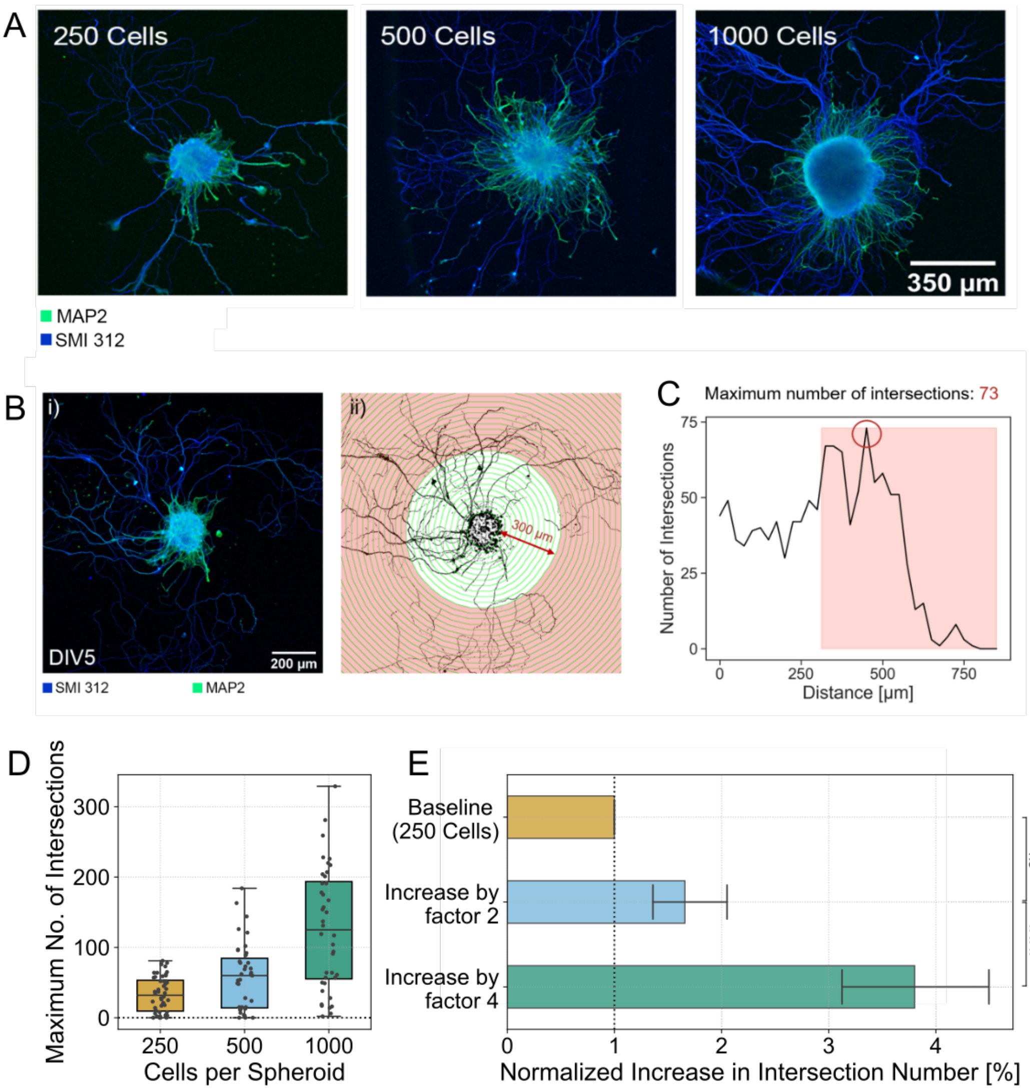

Figure S1. Correlation between retinal spheroid size and axon number.
(**A**) Representative fluorescence images of retinal spheroids with different cell sizes: 250 cells/spheroid, 500 cells/spheroid, and 1000 cells/spheroid. Immunostaining was performed using antibodies against MAP2 (green, labels dendrites) and SMI 312 (red, labels axons). (**B**) Illustration of the performed Scholl analysis counting the number of intersections at a radius of 300 µm. This radius should exclude most dendrites. (**Ci**) Quantification of the maximum number of intersections at 300 µm. The number of intersections does not equal number of axons but gives an estimate of the minimum number of axons. (**Cii**) Relative increase in the number of intersections relative to 250 cell spheroids.

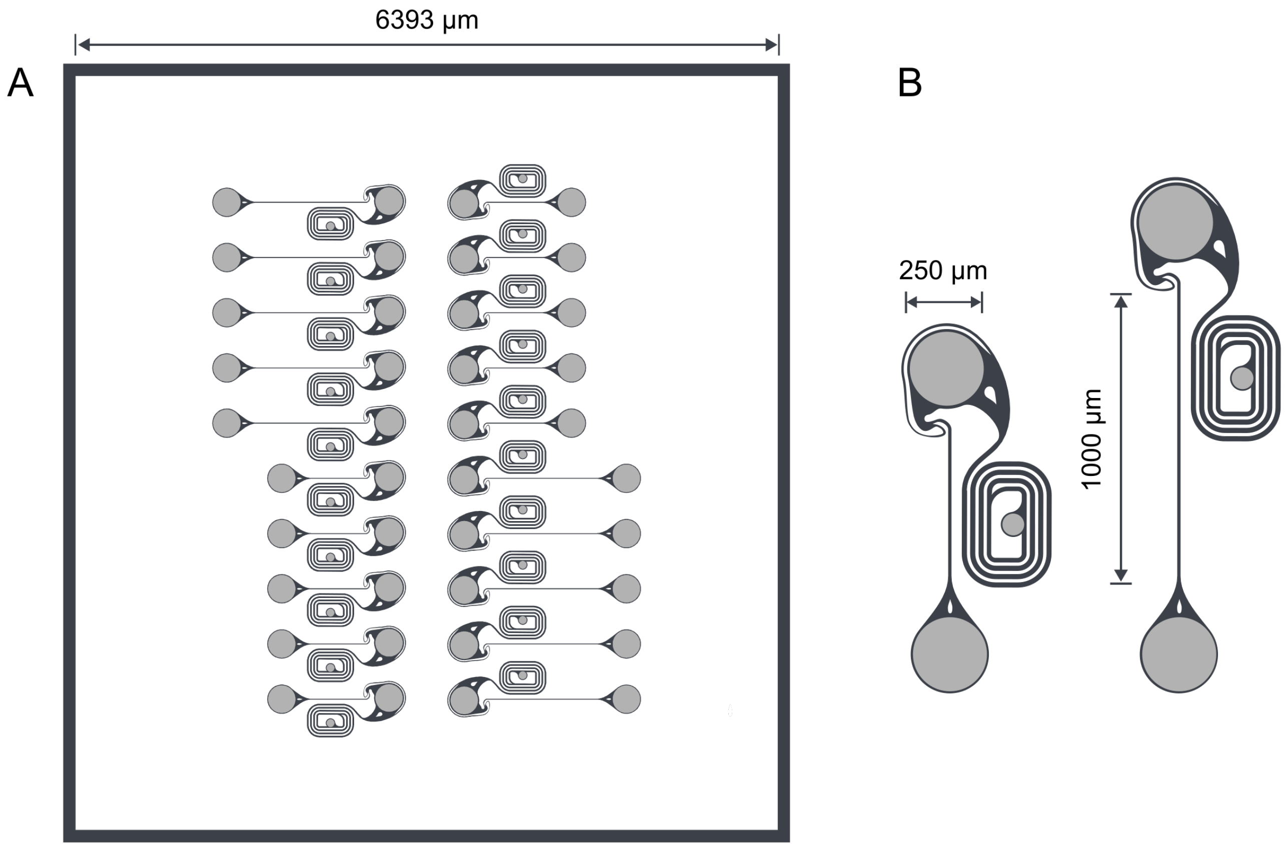

Figure S2. Microstructure designs with 1 and 0.5 mm channel length. (**A**) One microstructure fits 10 networks of the same channel length. (**B**) Only the channel length was varied between different microstructure architectures.

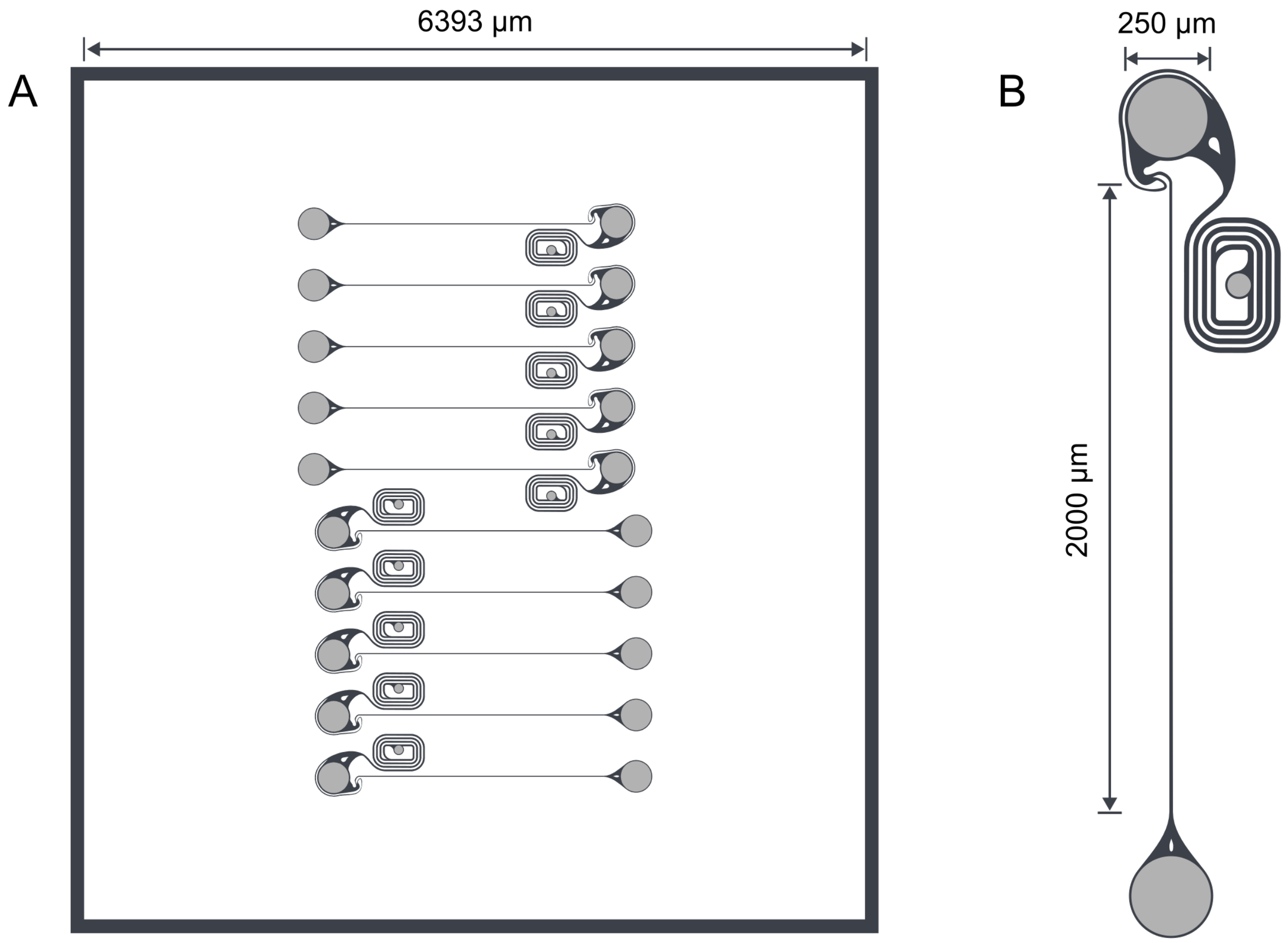

Figure S3. Microstructure designs with 2 mm channel length. (**A**) One microstructure fits 10 networks of the same channel length. (**B**) Only the channel length was varied between different microstructure architectures.

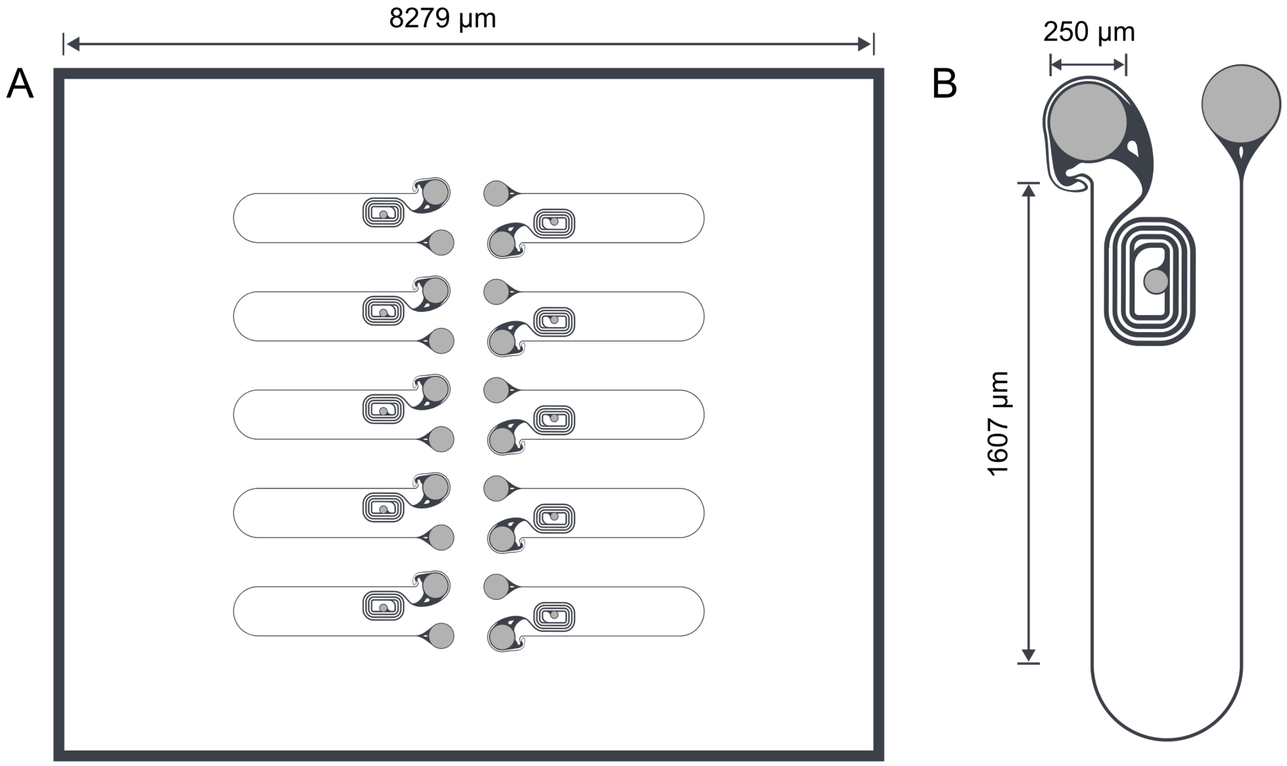

Figure S4. Microstructure designs with 4 mm channel length. (**A**) One microstructure fits 10 networks of the same channel length. (**B**) Only the channel length was varied between different microstructure architectures.

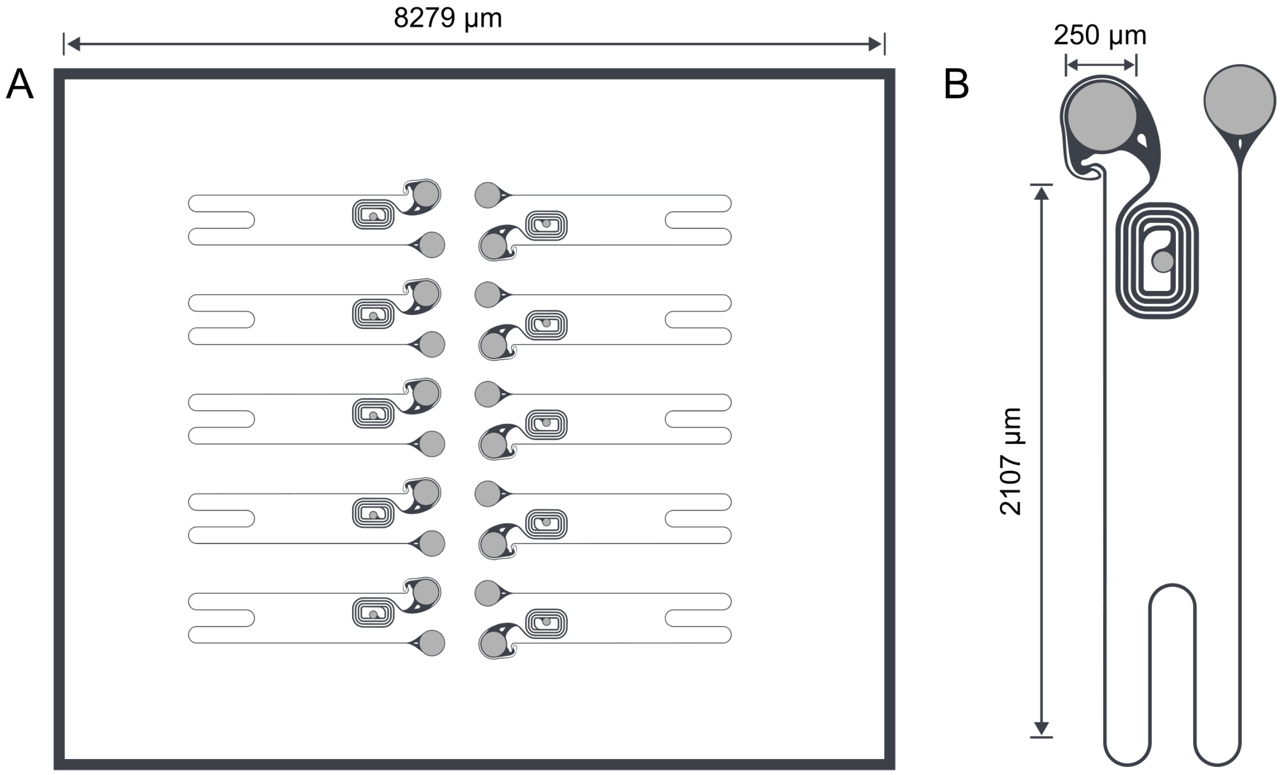

Figure S5. Microstructure designs with 6 mm channel length. (**A**) One microstructure fits 10 networks of the same channel length. (**B**) Only the channel length was varied between different microstructure architectures.

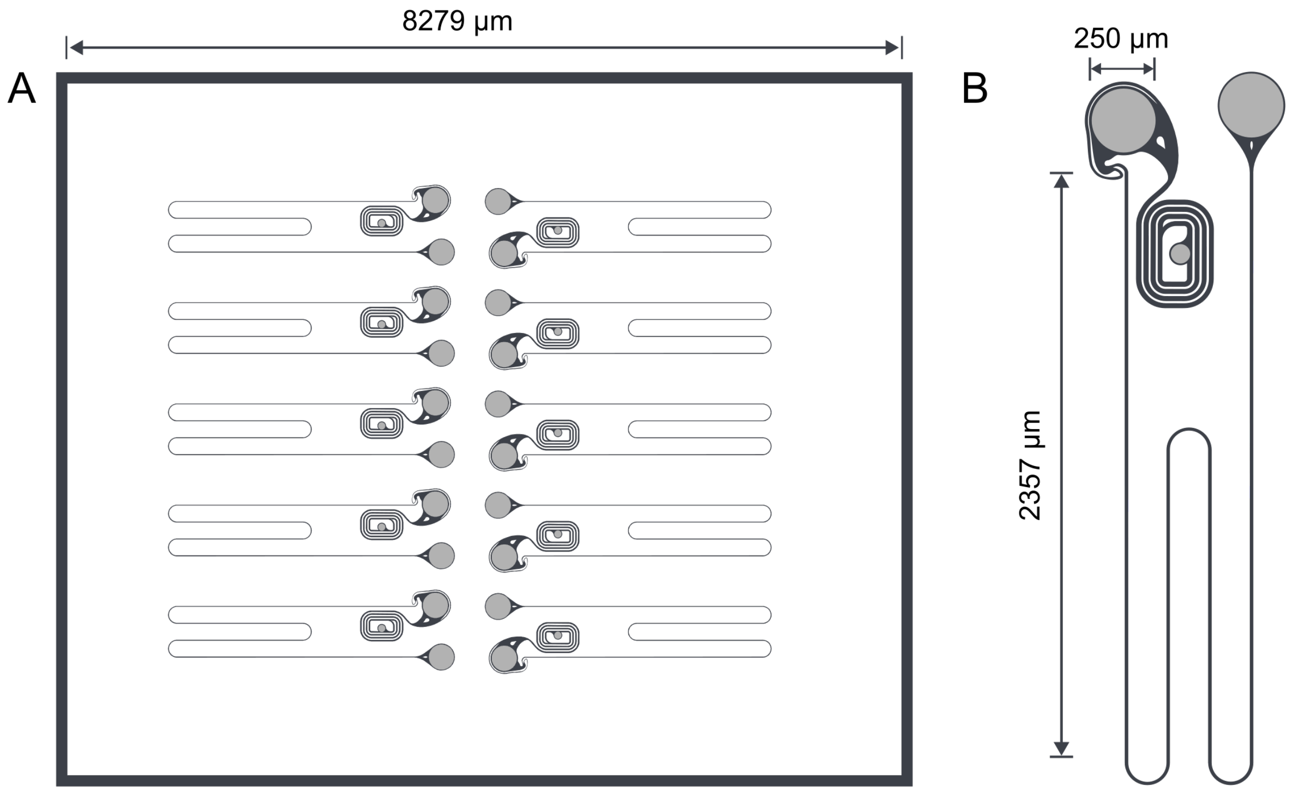

Figure S6. Microstructure designs with 8 mm channel length. (**A**) One microstructure fits 10 networks of the same channel length. (**B**) Only the channel length was varied between different microstructure architectures.

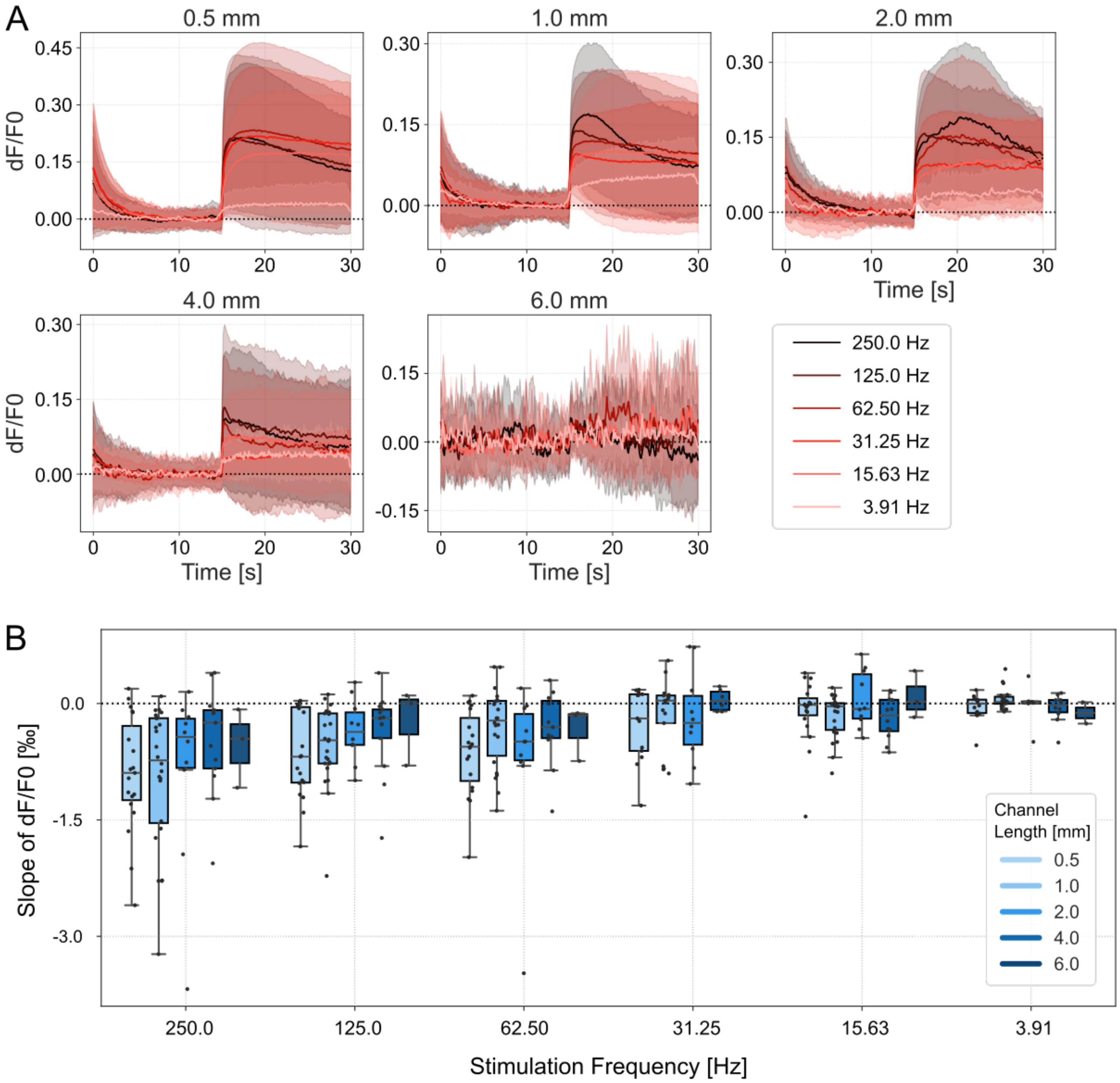

Figure S7. Thalamic response to continuous stimulation at varying frequencies.
(**A**) Average calcium traces of thalamic spheroids in response to axonal stimulation at different frequencies over increasing channel lengths. Stimulus onset at 15 s. The areas represent the estimated mean with a 95 % confidence interval. Error; SEM. ((**B**)) Calcium signal during continuous stimulation. Negative values indicate a negative slope and thus a decrease in fluorescence intensity over the stimulation period. A value close to 0 indicates a sustained response. Error bars, SEM.

2 SUPPLEMENTARY TABLES

Table S1: **MEA metrics used to quantify the spike trains.**

Metric	Definition
Percentage of intersected electrodes per network	An electrode was considered intersected if the axon bundle emerging from the source well crossed the electrode. The percentage of intersected electrodes per network was determined by dividing the number of intersected electrodes by the number of total electrodes in a network.
Morphological network integrity	A network was considered morphologically intact if mRuby expressing axons grew until the thalamic target. The morphological network integrity was defined as the percentage of morphologically intact networks per MEA and was calculated by dividing the number of intact networks by the total number of networks on a MEA.
Mean firing rate (MFR)	The firing rate was determined for each electrode by dividing the spike count by the total recording time. The mean firing rate of a network was derived by taking the mean over the network's electrodes.
Percentage of active electrodes per network	An electrode was considered active if its firing rate was above 1 Hz. The percentage of active electrodes per network was determined by dividing the number of active electrodes by the number of total electrodes in a network.
Functional network integrity	A network was classified as active if at least 75 % of its electrodes featured a firing rate above 1 Hz. The functional network integrity was defined as the percentage of active networks per MEA and was calculated by dividing the number of active networks by the total number of networks on a MEA.
Mean conduction speed per network	The conduction speed for forward propagating spike trains was determined from Spike-Triggered Time Histograms (STTH). STTH show the spike time latency distribution between two electrodes by plotting detected spikes on one electrode after a spike was detected on a triggering electrode. For each electrode, the spikes occurring within 16 ms after a spiking event were summed in bins of 0.05 ms. The mode of the distribution is used to define the conduction speed. The potential collision of two action potentials would not substantially affect this distribution, and therefore, it would not significantly impact the calculation of conduction speed. Only bins with more than six spikes were considered. The mean conduction speed between electrodes was calculated for each network. As the latency distribution requires at least two active electrodes, it could only be calculated for microstructures with longer or equal to 1 mm channels.

Percentage of forwards propagating spikes

A peak in the latency distribution of STTH exceeding the baseline level shows correlated spikes. The numbers of correlated spikes travelling from the proximal to the distal electrodes and from the distal to the proximal electrodes were determined to calculate the percentage of spikes conducted in the forward direction along the microchannel. The latency t_{Lat} between two spiking events was approximated based on the mean propagation speed and electrode spacing. The latency was then used to calculate a closed time interval T defined as $T = \left[t_{Lat} \times (1 - \alpha), t_{Lat} \times (1 + \alpha)\right]$ with a tolerance factor $\alpha = 0.5$. As t_{Lat} is dependent on the distance between the electrodes, the tolerance $t_{Lat} \times \alpha$ increases with the distance to account for the greater impact of deviations from the average conduction speed over longer distances. A successfully conducted spike in the forward direction was defined as a spiking event triggered at the proximal electrodes (closest to the source well) and measured within the time interval T at the electrodes closest to the target well. The percentage of forwards propagating spikes was then defined as $P_{Forwards} = \frac{N_{forwards}}{(N_{forwards}+N_{backwards})}$, with $N_{forwards}$ being the total number of successfully forwards conducted spiking events and $N_{backwards}$ the total number of backwards conducted spiking events.

Conduction fidelity

The conduction fidelity was defined as $C_{Fidelity} = \frac{N_{forwards}}{N_{detected}}$, with $N_{forwards}$ being the total number of successfully forwards conducted spiking events and $N_{detected}$ the total number of spikes detected at the proximal electrode. In other words, the conduction fidelity describes how many spikes starting from the retinal culture successfully reach the thalamic spheroid. Whenever possible, the conduction fidelity was calculated starting from both proximal electrodes to both distal electrodes and averaged to increase robustness. Networks with an MFR below 1 Hz in the proximal electrode were excluded as the spike number was too low to determine the conduction fidelity.

Table S2. Ordinary one-way ANOVA test results for the axonal outgrowth data. Summary of the test and p-value statistics of the ANOVA test and Tukey's multiple comparisons test for the relative increase in intersection number with spheroid size.

**Ordinary One-way ANOVA Test**						
P value	<0.0001					
P value summary	****					
F	32.58					
R squared	0.8445					
**Tukey's multiple comparisons test**						
Comparison	*Mean rank diff.*	*95% C.I.*	*Adjusted P*	*Summary*	*N1*	*N2*
250 vs. 500 Cells	-65.76	-162.6, 31.10	0.2075	ns	5	5
250 vs. 1000 Cells	-280.2	-377.1, -183.3	<0.0001	****	5	5
500 vs. 1000 Cells	-214.4	-311.3, -117.6	0.0002	***	5	5

Table S3. Kruskal-Wallis test results for the percentage of active electrodes per network. Test and p-value statistics of the Kruskal-Wallis and post-hoc Dunn's multiple comparisons test. The distributions of active electrodes per network were compared between the channel lengths.

**Kruskal-Wallis test**					
P value	<0.0001				
Exact or approximate P value?	Approximate				
P value summary	****				
Kruskal-Wallis statistic	115.9				
**Dunn's multiple comparisons test**					
Comparison	*Mean rank diff.*	*Adjusted P Value*	*Summary*	*N1*	*N2*
0.5 mm vs. 1 mm	-31.66	>0.9999	ns	99	132
0.5 mm vs. 2 mm	-42.29	>0.9999	ns	99	76
0.5 mm vs. 4 mm	44.21	>0.9999	ns	99	96
0.5 mm vs. 6 mm	101.3	0.0003	***	99	104
0.5 mm vs. 8 mm	160	<0.0001	****	99	112
1 mm vs. 2 mm	-10.63	>0.9999	ns	132	76
1 mm vs. 4 mm	75.87	0.0133	*	132	96
1 mm vs. 6 mm	132.9	<0.0001	****	132	104
1 mm vs. 8 mm	191.7	<0.0001	****	132	112
2 mm vs. 4 mm	86.5	0.0139	*	76	96
2 mm vs. 6 mm	143.6	<0.0001	****	76	104
2 mm vs. 8 mm	202.3	<0.0001	****	76	112
4 mm vs. 6 mm	57.08	0.2666	ns	96	104
4 mm vs. 8 mm	115.8	<0.0001	****	96	112
6 mm vs. 8 mm	58.71	0.1692	ns	104	112

Table S4. Kruskal-Wallis test results for the percentage of functionally intact networks. Summary of the test and p-value statistics of the Kruskal-Wallis and Dunn's multiple comparisons test for the distributions of functional network integrity for each channel length.

**Kruskal-Wallis test**					
P value	<0.0001				
Exact or approximate P value?	Approximate				
P value summary	****				
Kruskal-Wallis statistic	53.25				
**Dunn's multiple comparisons test**					
Comparison	*Mean rank diff.*	*Adjusted P Value*	*Summary*	*N1*	*N2*
0.5 mm vs. 1 mm	3.813	>0.9999	ns	16	16
0.5 mm vs. 2 mm	-0.4167	>0.9999	ns	16	12
0.5 mm vs. 4 mm	22.66	0.2147	ns	16	16
0.5 mm vs. 6 mm	38.94	0.0004	***	16	16
0.5 mm vs. 8 mm	50.63	<0.0001	****	16	16
1 mm vs. 2 mm	-4.229	>0.9999	ns	16	12
1 mm vs. 4 mm	18.84	0.6244	ns	16	16
1 mm vs. 6 mm	35.13	0.0022	**	16	16
1 mm vs. 8 mm	46.81	<0.0001	****	16	16
2 mm vs. 4 mm	23.07	0.3138	ns	12	16
2 mm vs. 6 mm	39.35	0.0012	**	12	16
2 mm vs. 8 mm	51.04	<0.0001	****	12	16
4 mm vs. 6 mm	16.28	>0.9999	ns	16	16
4 mm vs. 8 mm	27.97	0.0375	*	16	16
6 mm vs. 8 mm	11.69	>0.9999	ns	16	16

Table S5. Kruskal-Wallis test results for the spike conduction speed over time. Test and p-value statistics of the Kruskal-Wallis and post-hoc Dunn's multiple comparisons test. The conduction speed was compared between four weeks in culture.

**Kruskal-Wallis test**					
P value	<0.0001				
Exact or approximate P value?	Approximate				
P value summary	****				
Kruskal-Wallis statistic	43.23				
**Dunn's multiple comparisons test**					
Comparison	*Mean rank diff.*	*Adjusted P Value*	*Summary*	*N1*	*N2*
Week 1 vs. Week 2	-24.84	0.1139	ns	43	35
Week 1 vs. Week 3	-52.9	<0.0001	****	43	43
Week 1 vs. Week 4	-59.28	<0.0001	****	43	40
Week 2 vs. Week 3	-28.05	0.0484	*	35	43
Week 2 vs. Week 4	-34.44	0.0083	**	35	40
Week 3 vs. Week 4	-6.385	>0.9999	ns	43	40

Table S6. Friedman's test results for the percentage of active electrodes per network over the weeks *in vitro*. Summary of the test and p-value statistics of the Friedman's test and post-hoc Dunn's multiple comparisons test. The distributions of active electrodes per network at different time points (week 1 - 4) were compared. The test was computed separately for each channel length.

**Friedman's Test for each channel lengths**					
	Channel Length	*Friedman statistic*	*P value*	*Summary*	*N*
	0.5 mm	1	0.8013	ns	25
	1 mm	1.941	0.5847	ns	33
	2 mm	0.2019	0.9773	ns	19
	4 mm	7.788	0.0506	ns	24
	6 mm	14.45	0.0024	**	26
	8 mm	14.9	0.0019	**	28
**Dunn's multiple comparisons test for the 6 mm long microchannels**					
Comparison	*Rank sum diff.*	*Adjusted P Value*	*Summary*	*N1*	*N2*
Week 1 vs. Week 2	5	>0.9999	ns	26	26
Week 1 vs. Week 3	16.5	0.458	ns	26	26
Week 1 vs. Week 4	24.5	0.051	ns	26	26
Week 2 vs. Week 3	11.5	>0.9999	ns	26	26
Week 2 vs. Week 4	19.5	0.2172	ns	26	26
Week 3 vs. Week 4	8	>0.9999	ns	26	26
**Dunn's multiple comparisons test for the 8 mm long microchannels**					
Comparison	*Rank sum diff.*	*Adjusted P Value*	*Summary*	*N1*	*N2*
Week 1 vs. Week 2	11.5	>0.9999	ns	28	28
Week 1 vs. Week 3	21	0.1784	ns	28	28
Week 1 vs. Week 4	25.5	0.0498	*	28	28
Week 2 vs. Week 3	9.5	>0.9999	ns	28	28
Week 2 vs. Week 4	14	0.8838	ns	28	28
Week 3 vs. Week 4	4.5	>0.9999	ns	28	28

Table S7. Kruskal-Wallis test results for the effect of channel length on spike conduction speed. Test and p-value statistics of the Kruskal-Wallis and post-hoc Dunn's multiple comparisons test. The conduction speed was compared between neuronal networks in microstructures of different channel lengths.

**Kruskal-Wallis test**					
P value	0.0059				
Exact or approximate P value?	Approximate				
P value summary	**				
Kruskal-Wallis statistic	12.47				
**Dunn's multiple comparisons test**					
Comparison	*Mean rank diff.*	*Adjusted P Value*	*Summary*	*N1*	*N2*
1 mm vs. 2 mm	4.297	>0.9999	ns	67	40
1 mm vs. 4 mm	21.48	0.1526	ns	67	36
1 mm vs. 6 mm	38.13	0.0121	*	67	18
2 mm vs. 4 mm	17.18	0.647	ns	40	36
2 mm vs. 6 mm	33.84	0.0623	ns	40	18
4 mm vs. 6 mm	16.65	>0.9999	ns	36	18

Table S8. Kruskal-Wallis test results for the percentage of forwards propagating spikes in dependence on channel length. Test and p-value statistics of the Kruskal-Wallis and post-hoc Dunn's multiple comparisons test. The percentage of forwards propagating spikes was compared between neuronal networks in microstructures of different channel length.

**Kruskal-Wallis test**					
P value	<0.0001				
Exact or approximate P value?	Approximate				
P value summary	****				
Kruskal-Wallis statistic	67.26				
**Dunn's multiple comparisons test**					
Comparison	*Mean rank diff.*	*Adjusted P Value*	*Summary*	*N1*	*N2*
1 mm vs. 2 mm	41.42	<0.0001	****	75	52
1 mm vs. 4 mm	75.97	<0.0001	****	75	40
1 mm vs. 6 mm	73.33	<0.0001	****	75	19
2 mm vs. 4 mm	34.55	0.0117	*	52	40
2 mm vs. 6 mm	31.91	0.1485	ns	52	19
4 mm vs. 6 mm	-2.641	>0.9999	ns	40	19

Table S9. Kruskal-Wallis test results for the conduction fidelity in dependence on channel length. Test and p-value statistics of the Kruskal-Wallis and post-hoc Dunn's multiple comparisons test. The conduction fidelity was compared between neuronal networks in microstructures of different channel length.

**Kruskal-Wallis test**					
P value	0.0019				
Exact or approximate P value?	Approximate				
P value summary	**				
Kruskal-Wallis statistic	14.91				
**Dunn's multiple comparisons test**					
Comparison	*Mean rank diff.*	*Adjusted P Value*	*Summary*	*N1*	*N2*
1 mm vs. 2 mm	11.82	>0.9999	ns	85	55
1 mm vs. 4 mm	32.64	0.0182	*	85	44
1 mm vs. 6 mm	45.13	0.0107	*	85	21
2 mm vs. 4 mm	20.82	0.4951	ns	55	44
2 mm vs. 6 mm	33.32	0.171	ns	55	21
4 mm vs. 6 mm	12.49	>0.9999	ns	44	21

Table S10. Kruskal-Wallis test results for the effect of the channel length on the peak evoked response. Test and p-value statistics of the Kruskal-Wallis and post-hoc Dunn's multiple comparisons test. The PER was compared between neuronal networks in microstructures of different channel length.

**Kruskal-Wallis test**					
P value	0.0143				
Exact or approximate P value?	Approximate				
P value summary	*				
Kruskal-Wallis statistic	12.46				
**Dunn's multiple comparisons test**					
Comparison	*Mean rank diff.*	*Adjusted P Value*	*Summary*	*N1*	*N2*
0.5 mm vs. 1 mm	-0.7252	>0.9999	ns	25	23
0.5 mm vs. 2 mm	3.396	>0.9999	ns	25	18
0.5 mm vs. 4 mm	22.05	0.0676	ns	25	14
0.5 mm vs. 6 mm	-18.16	>0.9999	ns	25	4
1 mm vs. 2 mm	4.121	>0.9999	ns	23	18
1 mm vs. 4 mm	22.78	0.0587	ns	23	14
1 mm vs. 6 mm	-17.43	>0.9999	ns	23	4
2 mm vs. 4 mm	18.66	0.3183	ns	18	14
2 mm vs. 6 mm	-21.56	>0.9999	ns	18	4
4 mm vs. 6 mm	-40.21	0.0364	*	14	4
